# New Books and New Editions

**Published:** 1893-01-07

**Authors:** 


### New Books and New Editions.

### PHYSIOLOGY MADE EASY.*

Clearness, precision, brevity, and excellence are the characteristics of this small text-book. Physiology is a delightful study, but difficult. The teacher of physiology who smooths away the difficulties of its study, and yet succeeds in leaving a clear knowledge and appreciation of its facts in the mind, is recognised by the student as a real friend in need. Mr. Parkyn does not argue ; he informs. The ordinary teacher of physiology is distressingly argumentative as well as overpoweringly learned ; and as the subject is one about which opinions differ as widely as they do about questions theological, the controversial teacher who cannot restrain his argumentative propensities generally makes his text-book about ten times longer and more difficult than it ought to be. But this is death to the brain of the student. Mr. Parkyn knows better. Of course, there ought to be works which contain not only all that is known of physiolgy, but all that is thought and speculated by different, and even opposing schools. But are these ponderous tomes the books to put into the hands of youthful neophytes ? The Church instructs her disciples more wisely. She has prepared for their use a brief, simple, and compendious catechism. She thus furnishes them with an intelligent and accreting framework, into which not only may new knowledge be packed, but to which it may vitally adhere, and from which in time it may spring and grow.

Such a catechism as we have described is Mr. Parkyn's volume. It is a primer, or first book, and is admirably suited to students of all classes, medical as well as popular. It forms also an exceedingly convenient " reminder " for the consulting-room table of the busy medical practitioner. It is, perhaps, desirable to justify this very decided approbation ; but even if it were not, certain extracts which we have marked are interesting and instructive in themselves, and will furnish such a taste of the quality of the volume as may induce many readers, to their own great advantage, to buy and study it.

Mr. Parkyn begins with life in its very lowest forms. Here is his description of the beginnings of life in the cell form. "As we descend the scale of life we find that the variety in the nature and structure of the constituent cells becomes less and less as the organism becomes less complex. The division of labour correspondingly diminishes. At last, when the lowest forms are reached, they are found to consist of only one cell, which, living an independent existence, carries on all the processes necessary to its life, and, in fact, all division of labour has disappeared. Such cells are not only anatomical, but also physiological, units. In their cellular structure there is thus a connection between the highest and the lowest, the most complex and the simplest forms of animal life. These remarks apply equally to plants. In the lowest forms of animal and plant-life we, therefore, have organisms consisting of a single cell, of a very minute mass of living matter or protoplasm, the life-changes and life-history of which can be brought under observation by the microscope. Any knowledge such observation and experiment can reveal to us concerning the functions or vital actions of these simple organisms is so much information regarding the nature of living matter in its simplest form. From the study of these simple organisms something may, therefore, be learnt regarding the fundamental properties of living matter which cannot fail to throw light upon the great problem of life, even of life as manifested in man himself."

From this rudimentary beginning of all life it is a long leap to the coagulation of the blood. This is a phenomenon of great significance in the daily history of all animal organisms above the level of the very lowest. The lowest animals have no blood, and no heart or blood vessels for carrying the circulating nutriment to every part. But man and all mammalian animals are furnished with a highly complex blood-circulating system ; the blood itself being the most fundamentally important constituent of the body. As the great Hebrew lawgiver said, "The blood is the life." Not only the physiologist, but the surgeon and the physician are keenly alive to the significance of all the conditions which influence the coagulation of the blood. This physiological phenomenon is thus described by Mr. Parkyn. "Soon after being shed (four m inutes) the blood begins to pass into a jelly-like mass, or to clot, a change (complete in about ten minutes) known as coagulation. As the clot becomes firmer, drops of a pale yellow liquid are exuded from it, until, after the lapse of several hours, a red jelly-like mass of the same shape as the containing vessel is surrounded by this liquid. The blood thus becomes separated into two parts : (1) a solid red clot, and (2) a pale yellow liquid, or serum. The clot results from part of the liquid plasma solidifying. This solidification (which is indefinitely deferred at 0° C., and occurs most readily at 45° C.) takes the form of excessively fine threads, which, interlacing, form a most delicate net-work, in the meshes of which are entangled the blood corpuscles. These solid threads are composed of a substance called fibres, and the network formed by them, gradually contracting, squeezes out the remaining liquid portion of the blood, which appears as the pale yellow drops previously mentioned, or the serum of the blood. A blood-clot, therefore, consists of fibrin and corpuscles."

A good many other most interesting descriptive pages which we had marked for extracts must be passed over. Almost everybody, however, is more or less interested in the nervous system ; not only in its chief organs, the brain and spinal cord, but also in its branches and their adaptation to all manner of sensory perceptions. By means of the nervous system we see, hear, feel, taste, and touch, as well as connect ourselves in other ways with the external world which is our temporary environment. The retina, for example, the seeing part of the eye, is merely the optic nerve expanded at its distal termination. A similar expansion in other nerves enables us to hear and smell. A brief description of the terminal part of the olfactory nerve, which is called the "end-organ," may furnish our last quotation. Says Mr. Parkyn, "The end-organ of smell is the simplest of all the end-organs of the special senses. It consists of the mucous membrane lining the upper part of the nasal cavity. By the inward projection of two curved bones (turbinate bones), the nasal cavity is divided into three passages. The olfactory nerve is distributed only to the mucous membrane lining the two upper of these passages. Here the fibres of the olfactory nerve are believed to end in peculiar spindle-shaped cells (olfactory cells) situated between the ordinary columnar epithelium of the mucous membrane. These cells, therefore, constitute the real end-organ of smell. The olfactory nerve fibres arise from the olfactory lobes, from which they pass to the nostrils by piercing the cribriform plate of the ethmoid bone. In order that they may excite the end-organ, odorous substances must be in the form of gas or vapour, or at least be conveyed in a gaseous medium."

These extracts, which might be multiplied indefinitely, justify, we think, the judgment announced at the beginning of this article, that Mr. Parkyn's little text-book is characterised by clearness, precision, brevity, and excellence. So far as the study of physiology can be made easy, the task has been accomplished in the present volume. It has our most cordial approval and recommendation.

* "Human Physiology." By E. A. Parkyn, M.A. (London: Allman and Son, Limited.)

